# Noise reduction in protein-protein interaction graphs by the implementation of a novel weighting scheme

**DOI:** 10.1186/1471-2105-12-239

**Published:** 2011-06-16

**Authors:** George D Kritikos, Charalampos Moschopoulos, Michalis Vazirgiannis, Sophia Kossida

**Affiliations:** 1 Bioinformatics & Medical Informatics Team, Biomedical Research Foundation of the Academy of Athens, Athens, Soranou Efesiou 4, GR-11527, Greece; 2 Department of Informatics, Athens University of Economics & Business, Athens, 76 Patision Street, GR-10434, Greece; 3 Pattern Recognition Lab, Department of Computer Engineering & Informatics, University of Patras, Patra, Rio, GR-26500, Greece; 4 Institut Télécom, Ecole de Télécom ParisTech, Département Informatique et Réseaux, Paris France

## Abstract

**Background:**

Recent technological advances applied to biology such as yeast-two-hybrid, phage display and mass spectrometry have enabled us to create a detailed map of protein interaction networks. These interaction networks represent a rich, yet noisy, source of data that could be used to extract meaningful information, such as protein complexes. Several interaction network weighting schemes have been proposed so far in the literature in order to eliminate the noise inherent in interactome data. In this paper, we propose a novel weighting scheme and apply it to the *S. cerevisiae *interactome. Complex prediction rates are improved by up to 39%, depending on the clustering algorithm applied.

**Results:**

We adopt a two step procedure. During the first step, by applying both novel and well established protein-protein interaction (PPI) weighting methods, weights are introduced to the original interactome graph based on the confidence level that a given interaction is a true-positive one. The second step applies clustering using established algorithms in the field of graph theory, as well as two variations of Spectral clustering. The clustered interactome networks are also cross-validated against the confirmed protein complexes present in the MIPS database.

**Conclusions:**

The results of our experimental work demonstrate that interactome graph weighting methods clearly improve the clustering results of several clustering algorithms. Moreover, our proposed weighting scheme outperforms other approaches of PPI graph weighting.

## Background

Computational methods that successfully predict protein complexes are of instrumental importance to our understanding of the functions that take place in a living cell. Moreover, these methods can be used to guide biological experiments in order to confirm predicted complexes, therefore resulting in a reduction in the cost of such experiments. Protein complexes in a living cell are of dynamic nature; their composition and cardinality vary according to cell state and the challenges set forth to the cell by its environment. Since the data provided by interaction-detecting experiments lack temporal information, the results provided by clustering the interactome graph depict only a snapshot of the dynamic composition of protein complexes.

The problem of clustering protein interaction networks lies in the data itself, since methods that produce high-coverage of the proteome introduce a significant amount of noise in the form of false-positive interactions. On the other hand, methods that produce highly reliable interaction data, suffer from poor coverage of the complete interactome graph. This is known as the problem of noise inherent in protein interaction data, which is a drawback of the methods that detect protein interactions [1-4].

Protein-protein interaction networks are represented as undirected graphs G(V,E), in which the nodes V denote the proteins, while the edges E correspond to interactions among them [5]. In the remaining manuscript, the terms graph edge and protein interaction will be used interchangeably.

The issue of assigning weights to parameters has long been a crucial issue in optimization and learning from data as this process reflects the importance of single variables or groups of them [6]. On the other hand parameter or variable weighting has been used in recent prominent works for clustering. For instance, in [7] a set of weighting schemes that allow for an objective assignment of importance on the values of a data set in the issue of categorical clustering. The authors report results as to which weighting schemes show merit in the decomposition of data sets.

Modha et al. present an abstract framework for integrating multiple feature spaces in the k-means clustering algorithm [8]. The paper by Modha et al. deals with the definition of the optimal weighting scheme that yields the clustering that simultaneously minimizes the average within-cluster dispersion and maximizes the average between-cluster dispersion along all the feature spaces. Using precision/recall evaluations and known ground truth classifications, they empirically demonstrate the effectiveness of feature weighting in clustering on several different application domains.

Several approaches to PPI graph weighting make use of graph-theoretic methods (such as the Czekanowski-Dice distance [9]). However, these approaches make use only of the topology of the graph to induce weighting. In PPI data, on the other hand, there is an abundance of information that can be used to assess the reliability of an interaction. Examples of such information include the number of experiments, as well as the types of experimental procedure(s) used to detect the interaction [10-12].

In this manuscript, we present a novel noise reduction method by weighting protein interactions assigning higher score to the most probable true ones and a lower score to the ones derived as artifacts of the used detection method. Experiments were carried out using the yeast subset of the iRefIndex database [13] and results were compared to those of a recent topology-based weighting scheme [14]. In order to draw safe conclusions, four different clustering algorithms were applied (see Methods) and results were analyzed.

## Methods

The yeast subset of the iRefIndex database [13] is the dataset we used for all the analyses performed in this work. The iRefIndex database results from the union of six different interactome databases [15-20] after the redundancy has been removed. At the same time the iRefIndex database aggregates information such as the total number of experiments that report an interaction, experimental methods used, etc; information valuable to PPI graph weighting methods.

The majority of the clustering procedures consist of two steps: First, each binary protein interaction is assigned a weight, reflecting the confidence that this interaction is a true positive interaction. Then, the results are clustered using several different clustering algorithms. We performed experiments using all possible combinations of four different weighting schemes (described below) and four clustering algorithms.

### Graph Weighting Schemes

#### Graph theoretic methods

Brun *et al*. propose the PRODISTIN method of functional clustering of proteins based on the principle that the higher the number of common interactors shared by two proteins, the more likely they are to be functionally related [9]. In their contribution, the distance of every protein to their 1^st ^degree and 2^nd ^degree neighbors is calculated using the Czekanowski-Dice distance (CD-distance). More recently, Liu *et al*. proposed the Adjust-CD [14] weighting method. The Adjust-CD method is derived from the CD-Distance weighting method [9] and consists of an iterative procedure that relies solely on the network topology to calculate the reliability of a binary protein interaction. Interestingly, the Adjust-CD scoring method may also be used to discover protein interactions that do not originally exist in the protein interaction network. Of the recently proposed graph theory based weighting methods, we chose to apply the Adjust-CD method to the interactome of S.cerevisiae and evaluate the results.

#### Weighting using Gene Ontology

Lubovac et al. introduce two PPI graph weighing schemes [21,22] that calculate the weight of each interaction in the graph as the similarity featured by the Gene Ontology (GO) [23] terms of the corresponding proteins. We note that the similarity measures defined by Lubovac et al. focus on the overall similarity between each pair of proteins by calculating the average of pairwise GO term similarity values. Cho et al. also use semantic similarity information to infer the reliability of protein interactions [24]. The method of Cho et al., however, calculates the amount of information conveyed by each GO term and uses only the most informative terms to infer the weight of an interaction.

#### Weighting by experiment type

Von Mering *et al*. [1] recognize that each experiment type presents certain biases. For example, the well-known yeast-two-hybrid method is known to mainly detect interactions that take place in the nucleus of a cell, while purified complexes based methods detect only a subset of interactions between sensing related proteins [1]. According to von Mering *et al*., interactions detected by different experimental methods provide a set of interactions that is more accurate, even if the coverage of the interactome is rather limited.

Similarly, Nabieva *et al*. calculate the reliability of an experimental source by computing the fraction of interacting proteins that share the same function [11]. The weighting scheme implemented by Nabieva *et al*. assigns to each interaction a weight that is calculated as a function of the reliability of the experiment that reports the interaction.

In a more recent contribution, Pinkert *et al*. separate experimental methods in 3 categories: *in vivo, in vitro *and yeast-two-hybrid methods. Subsequently, they assign weights to interactions according to the combination of experimental categories by which the interaction was detected [12].

Pereira - Leal *et al*. also apply a weighting scheme, which does not distinguish between high- or low-throughput experiments, but boosts interactions that are detected in different experimental methods [10].

**Figure 1:**
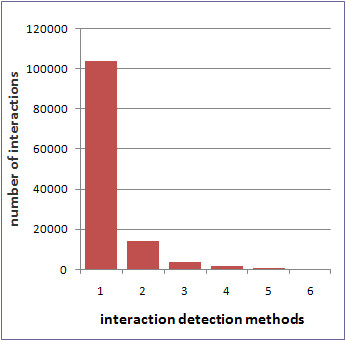
**Interaction frequency per number of detection methods**. Number of interactions per number of reporting detection methods in the iRefIndex database [13].

Using information derived from the work of von Mering *et al*. [1], we define the Experiment Type Weighting (ETW) scheme, which empirically assigns weights to interactions using the following procedure: If a protein-protein interaction is detected in only one experimental method, the corresponding graph edge is assigned a weight of 0.2. The ETW scoring scheme assigns a higher confidence level (0.6) to interactions that are verified by more than one type of experiment. In the rare case (see Figure 1), where an interaction is verified by 3 or more types of experiments, we assign a score of 1.0, reflecting the strong possibility that this is a true positive interaction.

#### Weighting by experiment plurality

Below we present two PPI graph weighting schemes that use the concept of experiment plurality, which is defined as the number of protein interactions reported by an interaction detection experiment. The first one is a novel weighting scheme, called MV scoring, and it is the main contribution of the present manuscript. The second one, called Simple Scoring, is based on the basic assumption that most scoring schemes use and it is used purely for evaluation purposes.

##### MV scoring

**Figure 2:**
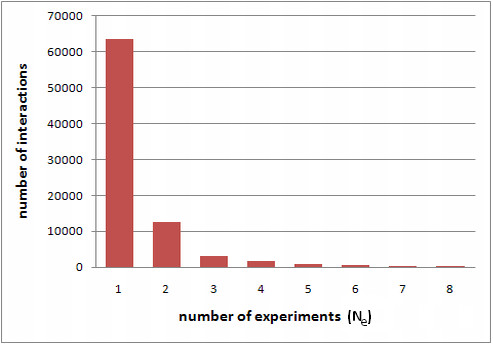
**Interaction frequency per number of experiments**. Number of interactions per number of reporting experiments in the iRefIndex database [13].

The proposed weighting scheme MV (from Michalis Vazirgiannis) takes into account the statistical distribution of the number of experiments (*Ne*) that report an interaction (see Figure 2) and the plurality of the experiment. We define the following edge weight assignment formula (Equation 1):(1)

Equation 1: MV weight assignment formula

The higher the number of experiments that report an interaction, the higher the weight the corresponding graph edge is assigned. On the other hand, the higher the pluralities of the experiments that report the interaction, the smaller the calculated confidence score. However, if a single experiment that reports the interaction has a low plurality (i.e. it is a high confidence experiment), the interaction will be assigned a high confidence score using the above formula (Equation 1).

The user-assigned factor ***α ***affects only interactions reported by more than one experiment (*Ne *> 1). A higher value of ***α ***conveys higher confidence levels to such interactions, whereas a smaller value tends to give the relative advantage to interactions that are reported by one experiment, with smaller plurality number. The value producing the best results has been experimentally adjusted to *α *= 2.

##### Simple Scoring

Purely for evaluation purposes, we define a naïve weighting scheme, which assigns a weight to an interaction based on the number of experiments (Ne) reporting the interaction, and the plurality of each one of those experiments. Based on the fact that high-throughput experiments are prone to detect false-positive interactions [1-4], if the interaction at hand is detected by a high-throughput (high plurality) experiment, it is empirically assigned a low score (i.e. 0.2 of max 1.0). On the contrary, low plurality experiments are generally thought to contain high confidence data, therefore an interaction detected by such an experiment will be assigned a high score (i.e. 0.9). The case where an interaction is detected by more than one experiments is of great statistical importance (see Figure 2) and is assumed to take place only for interactions that have high chances of being true-positives. As a result, we assign to these interactions a score that varies according to the pluralities of the overlapping experiments from 0.7 to 1.0.

### Clustering Algorithms

A number of algorithms have been proposed for clustering protein interaction networks. In order to cluster the weighed PPI graph, we only used the algorithms that support weighted graphs, such as MCL [25], the CMC [14] and the 2 Spectral methods described below. The RNSC algorithm [26], as well as more recent algorithms, such as COACH [27] could not be applied to a weighted PPI graph, since they do not take edge weights into account. However, for the sake of comparison, we include results of these algorithms applied to the unweighted PPI graph.

The RNSC algorithm [26] searches for a low cost clustering by composing first an initial random clustering, then iteratively moving one node from one cluster to another in a randomized fashion to improve the clustering cost. In order to avoid local minima, RNSC makes diversification moves and performs multiple experiments. Furthermore, it maintains a tabu list that prevents cycling back to a previously explored partitioning. Due to the randomness of the algorithm, different runs on the same input data produce different outputs.

The COACH algorithm [27] first detects dense subgraphs, as maximal sets of connected vertices whose degrees are greater than the network average. These subgraphs form the core of a candidate protein complex. Subsequently, the core is expanded by attaching nodes which are connected to nodes of the core by more than half of their edges. Note that the COACH algorithm is capable of detecting overlapping complexes, by assigning a protein to multiple clusters.

The Markov Clustering algorithm (MCL) [25] finds cluster structures in graphs by deterministically computing the probabilities of random walks. The starting point of the MCL algorithm is to build a Markov transition matrix, which captures the concept of random walks in a graph. The algorithm then iterates between expansion and inflation operators to the Markov matrix. Expansion computes random walks of higher length. In these walks, there is a high probability that a pair of nodes is on the same cluster. Inflation step, on the other hand, promotes intra-cluster walks and demotes inter-cluster walks. By pruning "weak" edges in the graph and simultaneously promoting "strong" edges, the algorithm discovers the cluster structure in the graph.

More recently Liu, Wong, and Chua proposed the Clustering on Maximal Cliques algorithm (CMC) [14]. CMC first detects all maximal cliques in the protein interaction network. The discovered cliques are then ranked according to their density, or weighted density if the edges of the graph are weighted according to a weighting scheme. Subsequently, cliques that feature a high degree of overlap are either removed or merged according to their interconnectivity. We note here that Liu *et al*., propose and use the Adjust-CD [14] weighting scheme (see below for a description) for the second step of their method. In the current contribution, we present an application of the CMC algorithm not only with Adjust-CD but also using other weighting schemes.

Additional experiments were carried out by using two variations of a Spectral Clustering algorithm. Using the Ng *et al*. spectral graph decomposition [28], we map the set of nodes in a graph to a set of points in the k-dimensional space. The number of dimensions (k) equals the number of clusters, which is discovered using the Eigengap heuristic [29].

Following the spectral decomposition, we apply to the set of resulting points two clustering algorithms, the recently proposed Kmeans++ algorithm [30] and the well known EM algorithm [31]. Using Kmeans++ in our experiments, we noticed a remarkable acceleration compared to the K-means algorithm. Since Kmeans++ is a randomized algorithm, each clustering experiment was repeated 30 times, of which we keep the clustering result featuring the minimum sum of square error. On the other hand, the EM algorithm can perform soft clustering assignment by calculating the probability of a protein belonging to each cluster. In this study, we assign each protein only to the most probable cluster. To the best of our knowledge, this is the first application of such variants of spectral clustering to the problem of protein interaction graph clustering.

Using a different approach, Bu *et al*. [32] proposed a spectral method to discover quasi-cliques, which are areas of a graph that exhibit high interconnectivity. Bu *et al*. extract such information using the eigenvalues of the decomposed graph and then sort the quasi-cliques according to their interconnectivity.

A more detailed report of the clustering algorithms used in PPI clustering is beyond the scope of this paper. A recent evaluation of clustering methods is given in [33].

## Results and Discussion

**Figure 3:**
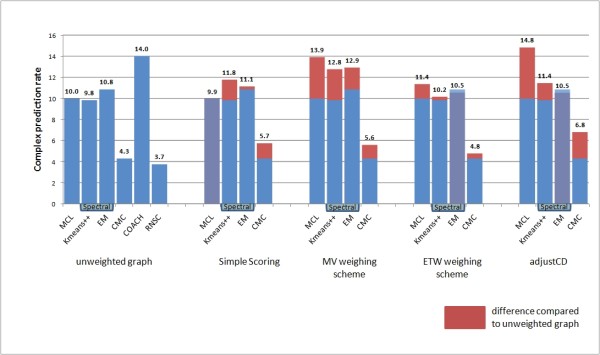
**Protein complex prediction rate chart**. Prediction rate results of all tested clustering algorithms versus all tested graph weighting schemes (the RNSC [26] and COACH [27] algorithms do not accept weighted input graphs).

**Figure 4:**
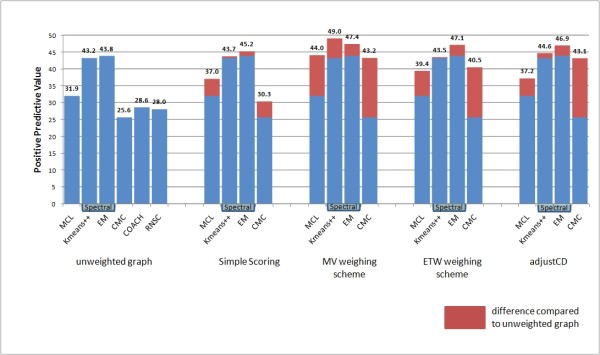
**Positive predictive value chart**. Positive predictive value results of all tested clustering algorithms versus all tested graph weighting schemes (the RNSC [26] and COACH [27] algorithms do not accept weighted input graphs).

**Figure 5:**
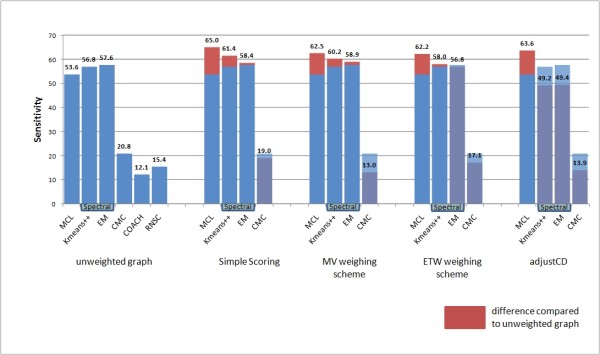
**Sensitivity chart**. Sensitivity results of all tested clustering algorithms versus all tested graph weighting schemes (the RNSC [26] and COACH [27] algorithms do not accept weighted input graphs).

**Figure 6:**
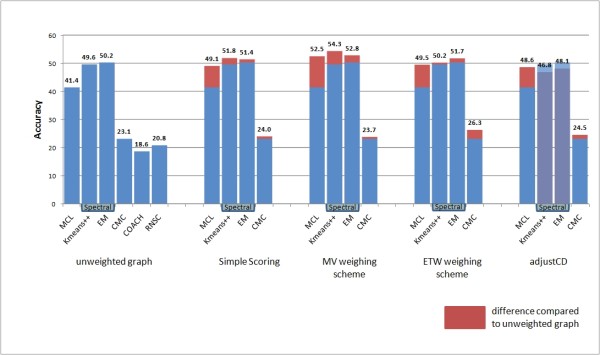
**Accuracy chart**. Accuracy results of all tested clustering algorithms versus all tested graph weighting schemes (the RNSC [26] and COACH [27] algorithms do not accept weighted input graphs).

Eliminating the noise inherent in the data is of vital importance in order to produce a valid clustering result. This is especially true in the PPI clustering context, since interactome data feature much noise mainly in the form of false positive interactions. The results presented in Figures 3, 4, 5 and 6 make evident that the proposed weighting schemes effectively eliminate the noise, thus resulting in better clustering results.

In order to compare the clustering results, we validate them against the list of known complexes present in the MIPS database [34]. The identifiers of proteins present in known complexes were translated from ORF IDs to UniProtKB IDs [35] to perform the clustering validation. As in a recent PPI graph clustering algorithm comparison [36], we chose not to use the complete list of complexes, but a filtered version that contains only verified complexes. For details on the filtering performed, see [36].

The metrics used to compare the algorithms' performances are mainly derived from the work of Brohee *et al*. [36], namely *Positive Predictive Value, Sensitivity *and *Accuracy*. We also use a prediction rate metric which is defined as the percentage of predicted clusters that match known complexes [37].

As far as the complex prediction rate is concerned, all tested algorithms benefit from the application of the MV weighting scheme (see Figure 3). Specifically for the MCL algorithm, we observe a 39% increase in complex prediction performance when applying noise reduction using the MV weighting scheme. The same algorithm, however, features an astonishing 49% increase in complex prediction rate when applying the Adjust-CD weighting method. What is more, the complex prediction performance of the COACH algorithm is higher than any other algorithm in the unweighted PPI graph, surpassed only by the MCL algorithm applied to an Adjust-CD weighted graph.

In the biological context, a high Positive Predictive Value (PPV) rating for a clustering algorithm would mean that the algorithm predicts protein complexes in which every protein belongs to the matching confirmed complex. High PPV value (see Figure 4) excludes the chance that the algorithm predicts complexes with proteins foreign to the corresponding confirmed complexes; it does not, however guarantee that every protein in the confirmed complex is present in the predicted one.

The above issue is addressed using the Sensitivity metric (see Figure 5). High sensitivity values guarantee that every protein in a confirmed complex is present in the matching predicted complex. This, however, does not certify that the predicted complex carries only proteins that belong to the confirmed complex.

From Figure 4 we see that the best results in PPV metric are obtained when applying either the MV weighting scheme or the Adjust-CD weighting scheme. Concerning the PPV results of the CMC algorithm, we observe an increase of 58% - 69% when applying either one of the MV, ETW or Adjust-CD weighting schemes. Moreover, using the MCL clustering algorithm in conjunction with a weighted graph using the MV weighting scheme we observe a 37.8% increase in Positive Predictive Value.

In fact, Positive Predictive Value is the only metric by which all algorithms are affected positively using graph weighting. This indicates that by assigning weights to the graph, the algorithms produce clusters that do not carry proteins irrelevant to the matching protein complex present in the MIPS reference set.

In the Sensitivity results chart (Figure 5), the MCL algorithm performs better than the other three algorithms in weighted graphs. This is contrary to the same results in the unweighted case; however, compared to other algorithms, MCL features a better increase in sensitivity when using weighted input.

As in all clustering methods, outstanding performance in precision (termed PPV in this context) cannot go without a loss of recall (sensitivity). This is evident when comparing the PPV and sensitivity rating of the CMC algorithm (see Figure 4 and Figure 5). By adding weights to the interactome graph, the CMC algorithm discovers smaller clusters each one being a subset of a complex in the MIPS reference set [34].

The price for this, however, is that the detected clusters lack several proteins that their corresponding reference set complexes feature. This holds also true for the results of Spectral methods when using the Adjust-CD weighting and the combination of the EM algorithm and the ETW weighting. Moreover, in contrast to all other tested algorithms, the sensitivity rating of the MCL algorithm was not hindered by the use of the Adjust-CD weighting scheme.

The Accuracy results chart (Figure 6) summarizes both PPV and sensitivity charts, since it requires both high Positive Predictive Value and sensitivity values to achieve a high Accuracy value [36]. In the biological context, a clustering result that accomplishes a high Accuracy value contains predicted complexes that carry those and only those proteins contained in their matched confirmed protein complex.

Spectral methods feature the best results for the unweighted graph compared to other clustering algorithms, although their out-performance compared to the MCL algorithm is decreased when using weighted graphs. This is largely due to the beneficial effect of graph weighting to both the PPV and sensitivity performance of the MCL algorithm. Furthermore, the combination of spectral methods and Adjust-CD weighting is the only one that features worse results compared to the unweighted case. This is expected, taking into account the poor sensitivity performance of this combination of algorithms and weighting scheme (see Figure 5).

**Figure 7:**
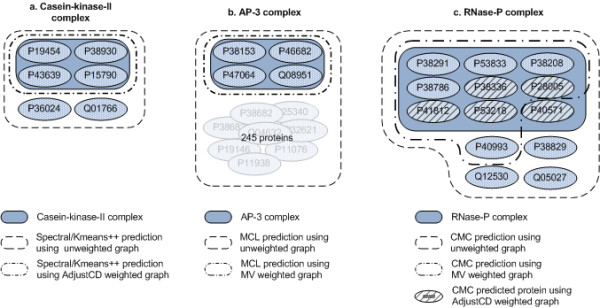
**Examples of clustering results**. Illustrated example exhibiting the differences between clustering results obtained by applying different weighing schemes to the original interactome graph.

In Figure 7a, the combination of an unweighted input graph with the Spectral-Kmeans++ algorithm results in a prediction of the complex "Casein-kinase-II" that lacks protein P15790 and also holds two artifactual proteins (P36024 and Q01766). By applying the Adjust-CD weighting scheme to the input graph, however, we observe a perfect prediction of the under question protein complex.

A stronger effect of interactome graph weighing is shown in Figure 7b. The prediction made by the MCL algorithm using an unweighted input graph detected all 4 proteins of the AP-3 complex, but falsely predicted that the same complex also holds more than 240 other proteins. Applying weights to the interactome graph according to the MV weighting scheme, the same algorithm made an exact prediction of the AP-3 complex.

In Figure 7c, the CMC algorithm successfully detected all 9 proteins of the RNase-P complex but also detected 5 non-relevant proteins when using the unweighted input graph. The combination of the Adjust-CD weighting with the CMC algorithm failed to detect 4 of the RNase-P proteins, but detected no non-relevant proteins. At the same time, by applying MV weighting, CMC discovered 8 out of 9 RNase-P proteins, while reporting only one additional protein (P40993). In fact, protein P40993 is a component of RNase-MRP, with which RNase-P is shown to share several subunits [38].

Summarizing, the combination of the MCL algorithm and the Adjust-CD features the best prediction rate and the second best sensitivity results. On the other hand, the combination of the Spectral-Kmeans++ algorithm with the MV weighting scheme features the best PPV and accuracy results. Taking all the above information into account, it becomes obvious that the best performing weighting schemes are the MV and the Adjust-CD ones. However, since the Adjust-CD one hinters the results of the spectral methods, we favor the use of the MV weighting scheme.

Finally, we note that a direct comparison between the CMC and the other clustering algorithms tested is not feasible, because the CMC algorithm, in contrast to MCL, Spectral-Kmeans++ and Spectral-EM algorithms, can assign a protein to more than one clusters. Arguably, this is a desirable property of a PPI clustering algorithm. However, applying the CMC algorithm to the current input data using the default parameters produced an exceptionally large number of clusters, which accounts for the lower performance compared to other algorithms.

## Conclusions

In this paper, we applied four different methods to introduce weights to the edges of the interactome graph according to the likelihood that an edge corresponds to a true positive interaction and conducted experiments using four different clustering algorithms.

The main contribution of this work is the development of a new weighting scheme, called MV scoring, which features superior noise reduction properties compared to known PPI weighting schemes.

As an outlook, we plan to further investigate the application of soft cluster assignment algorithms in protein interaction networks and assess the biological significance of our results.

## Competing interests

The authors declare that they have no competing interests.

## Authors' contributions

GK conceived the study, performed the experiments and wrote the paper. CM helped conducting the clustering experiments and aided revising the manuscript. MV conceived the MV weighting scheme and provided expert view on the clustering algorithms used and experiments conducted. SK guided the study and assisted in writing the manuscript. All authors read and approved the final manuscript.
